# Unplanned revision spinal surgery within a week: a retrospective analysis of surgical causes

**DOI:** 10.1186/s12891-016-0891-4

**Published:** 2016-01-15

**Authors:** Tsung-Ting Tsai, Sheng-Hsun Lee, Chi-Chien Niu, Po-Liang Lai, Lih-Huei Chen, Wen-Jer Chen

**Affiliations:** Department of Orthopaedic Surgery, Chang Gung Memorial Hospital, No. 5, Fusing St., Gueishan, Taoyuan 333, Linkou, Taiwan; Chang Gung University, College of Medicine, Taoyuan, Taiwan; Musculoskeletal Research Center, Chang Gung Memorial Hospital, Linkou, Taiwan

**Keywords:** Revision spinal surgery, Epidural hematoma, Inadequate decompression, Screw malposition, Posterior instrumentation

## Abstract

**Background:**

The need for revision surgery after a spinal surgery can cause a variety of problems, including reduced quality of life for the patient, additional medical expenses, and patient-physician conflicts. The purpose of this study was to evaluate the causes of unplanned revision spinal surgery within a week after the initial surgery in order to identify the surgical issues most commonly associated with unplanned revision surgery.

**Methods:**

We retrospectively reviewed the medical records of all patients at who received a spinal surgery at a regional medical center from July 2004 to April 2011 in order to identify those who required a revision surgery within one week of their initial surgery. Patients were excluded if they received a vertebroplasty, kyphoplasty, or nerve block surgery, because those surgeries are one-day surgeries that do not require hospital admission. In addition, patients with a primary diagnosis of wound infection were also excluded since reoperations for infection control can be expected.

**Results:**

The overall incidence of unplanned revision spinal surgery during the time period covered by this review was 1.12 % (116/10,350 patients). The most common surgical causes of reoperation were screw malposition (41 patients), symptomatic epidural hematoma (27 patients), and inadequate decompression (37 patients). Screw malposition was the most common complication, with an incidence rate of 0.82 %. Screw instrumentation was significantly associated with revision surgery (*p* = 0.023), which suggests that this procedure carried a greater risk of requiring revision. The mean time interval to reoperation for epidural hematomas was significantly shorter than the intervals for other causes of revision spinal surgery (*p* < 0.001), which suggests that epidural hematoma was more emergent than other complications. Also, 25.93 % of patients who underwent hematoma removal experienced residual sequelae; this percentage was significantly higher than for other surgical causes of revision spinal surgery (*p* = 0.013).

**Conclusions:**

The results suggest that to avoid the need for reoperation, screw malposition, inadequate decompression, and epidural hematoma are the key surgical complications to be guarded against. Accordingly, adequate decompression, epidural hematoma prevention, and proper pedicle screw placement may help reduce the incidence of revision surgery.

## Background

Since most spinal surgeries are elective procedures that aim to improve quality of life, any unplanned revision surgery would obviously be highly undesirable [1]. Conflict can arise between the patient and surgeon if a revision surgery is required within a short period of time after the initial operation, and such conflict may even become an obstacle for further medical treatment.

Previous studies have reported that the incidence of significant spinal cord or cauda equina injury following spinal surgery ranges from 0 % to 2 % [2–4]. Researchers have also analyzed the etiology, risk factors, and long-term results of reoperation [5, 6]. However, no prior studies have analyzed the short-term results of an initial operation in order to ascertain the potential surgical causes of revision surgeries. According to a study by Gruskay et al., patients are typically discharged within one week after receiving an elective spinal surgery [6], so the clinical features of patients requiring a revision surgery within seven days after surgery would hold significant practical value. Relatedly, in our institution, as in many other hospitals, surgical assignments other than emergency surgeries are scheduled on a weekly basis. Each surgeon is assigned a specific operating room on a specific day of the week, so any revision surgery that a patient requires will effectively constitute an unplanned surgery for the surgeon in question. Thus, any surgical complications requiring an unplanned revision surgery within one week of an initial surgery also have practical implications with regard to the scheduling of surgical assignments. Accordingly, the purpose of this study was to evaluate the surgical causes of revision spinal surgery within one week after the initial spinal surgery, as well as the incidence rates of those causes, in order to investigate the association of those procedures with revision surgery and, in turn, to develop potential prevention strategies.

## Methods

We obtained approval from Spine Section, Department of Orthopaedic Surgery, Chang Gung Memorial Hospital, to get access to the medical records; in addition, this study was approved by the Institutional Review Board of Chang Gung Memorial Hospital, Taiwan. The medical records of all patients who underwent adult spinal surgery for degenerative disease, tumor, or fracture in the Department of Orthopedic Surgery of Chang Gung Memorial Hospital in Taoyuan, Taiwan, between July 2004 and April 2011 were reviewed. Non-admitted spine surgery patients, such as, for example, patients receiving vertebroplasty, kyphoplasty, or nerve block surgery, were excluded. In addition, patients who had a primary diagnosis of wound infection and received wound debridement were also excluded, because reoperations for infection control can be expected in such cases.

**Fig. 1:**
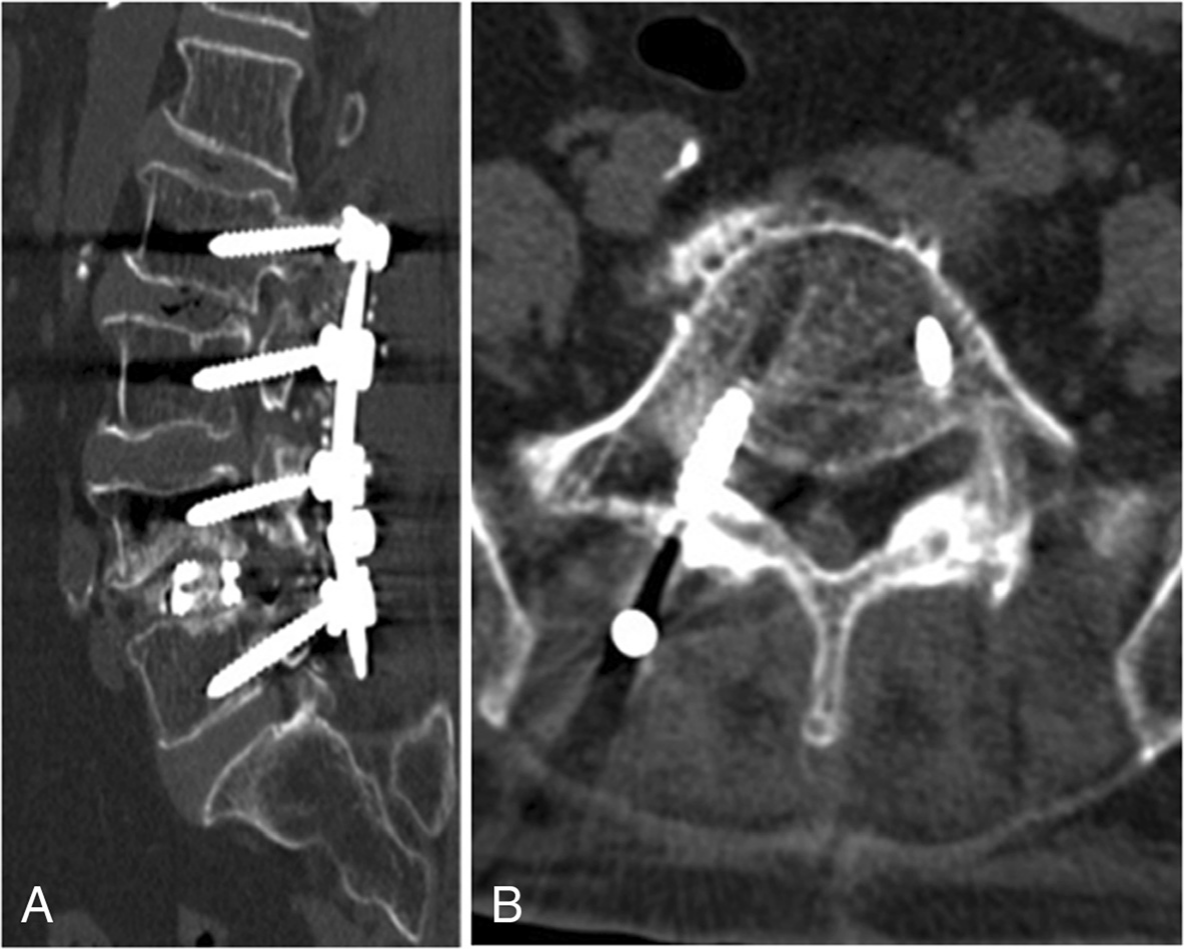
Computed tomography images of nerve root irritation. This patient had persistent pain in the right thigh and calf after L2–5 laminectomy, transpedicular screw fixation, and interbody fusion. **a** Sagittal and **b** cross-sectional postoperative computed tomography showed that the right L5 screw had been misplaced and cut out of the pedicle, causing right L5 nerve root irritation

**Fig. 2:**
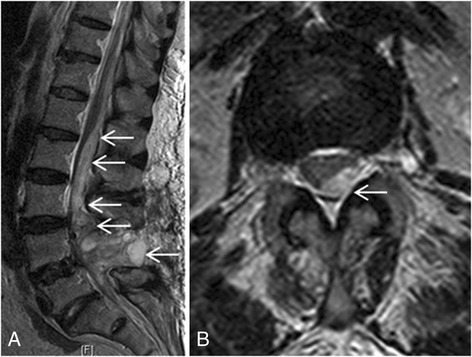
Magnetic resonance images of epidural hematoma and cauda equina syndrome. This patient had progressive peri-anal numbness and left lower extremity weakness on postoperative day 1. **a** Postoperative sagittal magnetic resonance imaging revealed epidural hematoma extending from L4–5 to the L2 level (arrows). **b** Cross-sectional magnetic resonance imaging revealed epidural hematoma at L2–3 (arrow) with compression-induced cauda equina syndrome

**Fig. 3:**
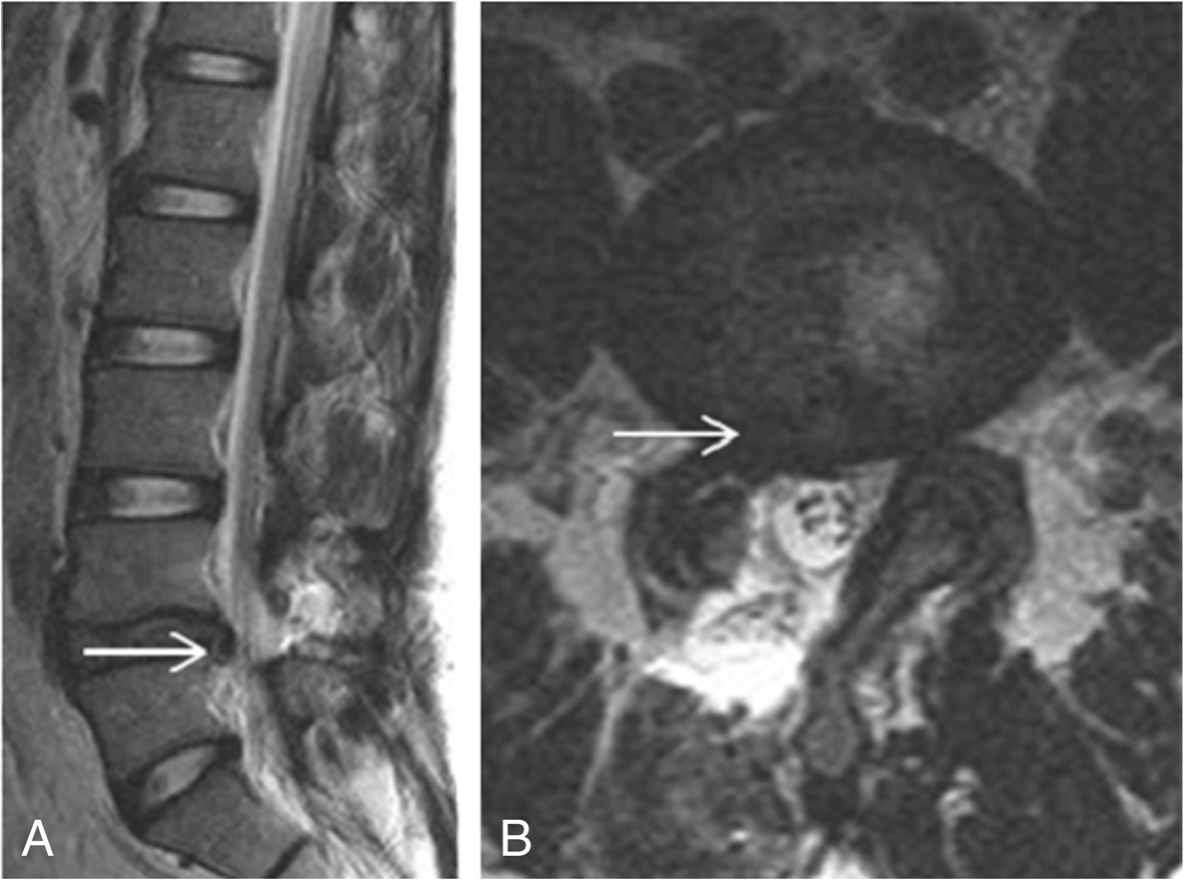
Magnetic resonance images of persistent herniated disc with dural sac compression. This patient had right buttock and leg pain for 3 months, and the symptoms persisted after right L4–5 discectomy. Postoperative **a** sagittal and **b** cross-sectional magnetic resonance imaging revealed persistent herniated disc with dural sac compression (*arrows*)

The etiology of revision surgery was investigated by clinical and imaging assessments, and confirmed by intraoperative findings during reoperation. The clinical outcomes were evaluated by neurological improvement of pain scale, numbness sensation, and muscle power. Preoperative and postoperative radiographic images were obtained. Computed tomography (CT) and/or magnetic resonance imaging (MRI) were additionally used to confirm a given diagnosis (Figs. 1, 2 and 3).

The following clinical outcome variables were compared to determine possible risk factors of revision spinal surgery: age, time interval between the initial surgery and revision surgery, length of hospital stay, number of inpatient operations, and presence of new post-operative neurological deficit.

### Statistical analysis

To analyze the differences between each surgical cause of revision surgery and their relationships with other clinical variables such as age, interval to reoperation, length of hospital stay, and neurological improvement, the one-way ANOVA and Fisher’s exact tests were used. Chi-square analysis was also used to demonstrate the relationships between posterior screw instrumentation procedure and each etiology of reoperation. All analyses were performed using SPSS, version 17.0 (SPSS Inc., Chicago, IL, USA). Significance was set at *p* < 0.05.

## Results

Out of the 10,350 patients reviewed, 8992 (86.9 %) surgeries were performed for degeneration, 459 (4.4 %) were performed for scoliosis, 658 (6.4 %) were performed for fractures, 220 (2.1 %) were performed for tumors, and 21 (0.2 %) were performed for various other reasons. The surgeries were further categorized according to the surgical location, as follows: cervical spine (n = 362, 3.5 %), thoracic spine (n = 331, 3.2 %), thoracolumbar spine (n = 572, 5.5 %), lumbar spine and sacrum (n = 9085, 87.8 %). Out of all 10,350 patients, a total of 116 patients (50 males and 66 females; mean age, 64.7 years; age range, 23–93 years) required revision surgery within seven days from their initial spinal operation. Among all 10,350 cases included, screw instrumentation was used in 4,984 patients during the initial surgery, while instrumentation was not used in the remaining 5,366 cases. In patients requiring revision surgery, the surgical site was located at the cervical spine in 2 cases (complication rate: 0.55 %, 2/362), at the thoracic spine in 11 cases (3.32 %, 11/331), at the thoracolumbar spine in 14 cases (2.45 %, 14/572), and at the lumbar and sacral spine in 89 cases (0.98 %, 89/9,085). The major surgical causes (in terms of frequency) of revision surgery were screw malposition (incidence rate: 0.82 %, 41/4,984), symptomatic epidural hematoma (0.26 %, 27/10,350), and inadequate decompression (0.36 %, 37/10,350). It should be noted that inadequate decompression indicates that the diagnosis and surgery were correct, but that the decompression achieved was insufficient, such that the patient’s symptoms persisted or got worse after the initial surgery. In such cases, more extensive decompression surgery is required. As such, the cause of any additional revision surgeries for patients in such cases was the same as the original cause of the index surgery. A number of less common causes accounted for the remaining 11 cases of revision surgery (0.11 %, 11/10,350). Specifically, the rare causes were as follows: wrong-level operation (one case), incidental dural tear with nerve root entrapment (one case), and broken drainage tube (two cases). In addition, six patients with unknown sciatica required secondary nerve root steroid injections, and one case involving a conus medullaris tumor required revision surgery. Drains were placed at the subfascial layer in almost all the index surgeries, with the exception of those cases in which there was very little bleeding.

There were no significant differences between each cause of revision surgery in terms of length of hospital stay, number of inpatient operations, or underlying medical conditions, including cardiovascular disease, respiratory disease, diabetes mellitus, cancer, smoking, and alcohol intake (Table 2).

### Screw malposition

Of the 116 patients who had revision surgeries, 68 (58.62 %) had undergone posterior screw fixation in the initial spinal surgery, while the remaining 48 (41.38 %) had received no instrumentation. These data indicate that screw instrumentation was significantly associated with the overall incidence of revision surgery (*p* = 0.023; Table 1). However, since only the patients who underwent screw instrumentation were subject to screw malposition, cases of screw malposition were subsequently excluded when analyzing the association between screw instrumentation and all other causes of revision surgery. Those results indicated that a lack of screw instrumentation, rather than the use of screw instrumentation, was significantly associated with all the other causes of revision surgery (*p* = 0.034; Table 1).

**Table 1:** The relationship between posterior screw instrumentation and each surgical cause of revision spinal surgery

	Instrumentation (*n* = 4984)	No instrumentation (*n* = 5366)	χ^2^	*p*
All causes of revision surgery	68	48	5.1472	0.023
All causes of revision surgery excluding screw malposition	27	48	4.4705	0.034
Screw malposition	41	0		n/a
Epidural hematoma	10	17	1.3402	0.247
Inadequate decompression	12	25	3.6765	0.055

For the 41 cases in which screw malposition was the cause of revision surgery, the mean time interval between the initial surgery and revision surgery was 6.20 days, the mean number of inpatient surgical procedures was 2.15 procedures, and the mean length of hospital stay was 15.76 days. In addition, two (4.88 %) of these 41 patients continued to have persistent neurological deficit despite revision surgery (Table 2). Of the 41 patients who required revision surgery for screw malposition, 11 patients underwent screw revision, while 30 patients underwent screw removal without re-insertion.

**Table 2:** Comparison of risk factors for revision spinal surgery between surgical causes

	Screw Malposition (*n* = 41)	Epidural Hematoma (*n* = 27)	Inadequate Decompression (*n* = 37)	Others (*n* = 11)	*p*
Age (y)	65.51 ± 12.02 (38–93)	67.30 ± 17.61 (24–88)	54.35 ± 11.35 (30–75) *	65.91 ± 15.66 (38–83)	<0.001
Interval to reoperation (d)	6.20 ± 1.40 (2–7)	2.70 ± 2.48 (0–7) *	5.27 ± 1.97 (1–7)	4.45 ± 1.81 (2–7)	<0.001
Length of hospital stay (d)	15.76 ± 7.65 (7–55)	23.11 ± 16.92 (5–74)	17.00 ± 13.47 (4–55)	21.73 ± 19.45 (7–60)	0.117
Number of inpatient operations	2.15 ± 0.48 (2–4)	2.30 ± 0.54 (2–4)	2.08 ± 0.60 (2–4)	2.09 ± 0.30 (2–3)	0.410
Presence of new post-operative neurological deficit	4.88 % (2)	25.93 % (7)*	2.70 % (1)	9.09 % (1)	0.013^a^

Data are presented as mean ± standard deviation (range) by one-way ANOVA, with the exception of presence of new post-operative neurological deficit, which is presented as % (number of cases)

^a^Fisher’s exact test

*p < 0.05

### Symptomatic epidural hematoma

Spinal instrumentation was not significantly associated with the occurrence of epidural hematoma (*p* = 0.247; Table 1). For the 27 cases in which epidural hematoma was the cause of revision surgery, the mean time interval between the initial surgery and revision surgery was 2.70 days, the mean number of inpatient surgical procedures was 2.30 procedures, and the mean length of hospital stay was 23.11 days. More specifically, it was observed that the mean time interval to reoperation for patients with epidural hematoma was significantly shorter than for other causes of revision spinal surgery (*p* < 0.001; Table 2), which likely reflects the urgency of treating epidural hematoma. In addition, while improvement of neurological deficits was observed in 20 (74.07 %) of these 27 patients, seven (25.93 %) patients continued to have persistent neurological deficit despite revision surgery, a rate that was significantly higher than those for other causes of reoperation (*p* = 0.013; Table 2).

### Inadequate decompression

Thirty-seven patients underwent revision surgery for inadequate decompression. The incidence of inadequate decompression among those who had posterior instrumentation was 0.24 % (12/4,984), while it was 0.47 % (25/5,366) for those who did not have posterior instrumentation. Although this difference was not statistically significant (*p* = 0.055), the result indicated that spinal instrumentation may be related to the lower incidence of inadequate decompression (Table 1).

For the 37 cases in which inadequate decompression was the cause of revision surgery, the mean time interval between the initial surgery and revision surgery was 5.27 days, the mean number of inpatient surgical procedures was 2.08 procedures, and the mean length of hospital stay was 17.0 days. Only one (2.70 %) of these 37 patients continued to have persistent neurological sciatica despite revision surgery. The mean age of the inadequate decompression group was younger than those of other groups (*p* < 0.001; Table 2), and the group was also predominantly male.

## Discussion

An unplanned revision surgery after a spinal operation is a highly objectionable outcome for a patient and his/her family. Accordingly, the need for revision surgery, in addition to resulting in higher medical care expenditures and higher risks of sequelae, may even jeopardize the patient-doctor relationship. Some previous studies have reported on the causes and risk factors of revision surgery in the long term after lumbar spine surgery [6, 7], while others have focused on the perioperative neurological complications of spinal surgery [8–10]. McCormack et al. took a more general view to evaluate all types of spinal surgeries performed over a three-year period at a single orthopedic specialty hospital in order to analyze the causes of unplanned readmissions within 30 days from last admission [11]. Bernatz et al. took an even more general approach in conducting a meta-analysis of 24 recent studies to determine the present rate of 30-day readmissions in orthopedics in general, as well as in various orthopedic subspecialties [12]. According to the nine spine-related studies included in their analysis, the current 30-day readmission rate for the spine subspecialty is 5.0 %. However, no previous studies have focused specifically on investigating surgical causes of complications requiring revision surgery within only one week after the initial operation.

In the present study, we found that the need for revision spinal surgery was relatively rare, with the overall incidence rate being 1.12 % (116/10,350 patients), which was roughly consistent with the 2.2 % reoperation rate reported by McCormack et al. [11]. In these rare cases requiring revision surgery, we found three surgical causes with relatively high frequencies of occurrence: screw malposition, symptomatic epidural hematoma, and inadequate decompression. In addition, a number of other direct causes for revision surgery were also found; however, each of them occurred far more rarely than the above mentioned three causes, with only a single case for each cause.

The major indication for early reoperation is a deterioration in neurological status [13]. If a patient experiences new-onset motor weakness and sciatica after spinal surgery, several possibilities should be taken into consideration: screw malposition, intraoperative nerve root injury, neuropraxia, and vascular compromise [14]. Spinal instrumentation has previously been reported to increase the risk of neurological injury [8, 15]. The potential risks and complications associated with it include nerve root irritation (0.2–1.1 %), fracture of the pedicle (0.5–1.1 %), and bending of the pedicle screws (0.1 %) [16, 17].

In a systematic review discussing the complications of treating pediatric scoliosis with screw fixation, Hicks et al. [18] stated that the most commonly reported complication of thoracic spine instrumentation was screw malposition, with a rate of 15.7 %. In our series, the malpositioned screws were placed either too inferiorly or too medially. The placement of pedicle screws is technically demanding and time-consuming. As such, patients who undergo spinal instrumentation have a greater chance of requiring revision surgery than patients who do not receive spinal instrumentation.

According to a review by Glotzbecker et al., the incidence rate of symptomatic spinal epidural hematoma ranges from 0 % to 1 % [19], a range that includes the rate of 0.26 % found in our study. The risk factors for spinal epidural hematoma identified in previous studies include multi-level procedures and preoperative coagulopathy [17, 20, 21]. Also, we have previously reported that preoperative diastolic blood pressure, intraoperative use of gelfoam for dura coverage, and postoperative drain output are risk factors with statistical significance for symptomatic epidural hematoma [22]. Other factors such as age, body mass index, durotomy, and the use of drainage tubes do not seem to influence the incidence.

Our data indicated that revision surgeries for symptomatic epidural hematoma are more urgent than those for other complications, having the shortest time interval to reoperation. Preoperative neurological status and time interval to reoperation are correlated with recovery outcomes. Lawton et al. [23] reported that 83 % of Frankel grade D patients recovered completely, compared to only 25 % of Frankel grade A patients. Also, better neurological recovery was achieved for patients who received surgical decompression within 12 h of symptom onset, as compared to patients who had an identical preoperative Frankel grade and for whom revision surgery was delayed beyond 12 h. The findings of Lawton’s study were consistent with the results of our previous study [24], so it would be reasonable to conclude that for those cases in which symptomatic epidural hematoma does occur after operation, rapid diagnosis and urgent surgical evacuation are required to achieve better neurological recovery.

Neural element decompression plays an important role in spinal surgeries by relieving pain and providing an optimal environment for neurological recovery. However, it is not always successful and it may further destabilize the spine. Inadequate decompression, whether resulting from a limited laminectomy or an incomplete discectomy, often leads to undesirable outcomes.

Guigui et al. [25] reported that 56 % of patients who underwent reoperation had inadequate decompression; they thus concluded that preoperative planning of the neural decompression should be carried out to achieve good surgical results and avoid the need for revision surgery.

Spinal decompression involves the release of pressure on the spinal cord and nerve roots, and the removal of osteophytes, hypertrophic ligaments, or protruding discs. However, it is often accompanied by the loss of spinal stability due to the destruction of the aforementioned structures. In our study, inadequate decompression occurred more frequently in patients who did not receive spinal instrumentation. This is most likely because the surgeons tended to ensure sufficiently wide decompression for instrumentation and fusion procedures due to the possibility of spine destabilization, in contrast to instances of relatively narrow decompression that may have occurred when instrumentation and fusion were not performed.

It is notable that while past studies that have investigated the causes of unplanned revision surgery within 30 days have found wound infections to be the most common cause, the current study found no instances in which wound infection was the cause of an unplanned revision surgery within one week of the initial surgery. Naturally, this simply reflects, to a large extent, the different speeds with which different complications cause problems requiring a revision surgery (the symptoms of wound infections, for example, typically do not develop within a week), but we believe that highlighting these differences between causes of reoperation within one week versus the causes within one month will be of practical use and reference to surgeons as they monitor their patients post-operatively. That is, the results of this study, combined with past studies covering longer post-operative timeframes, will give surgeons a better idea of the problems that are most likely to occur within one week, as well as which potential issues may need to be monitored more closely thereafter.

**Fig. 4:**
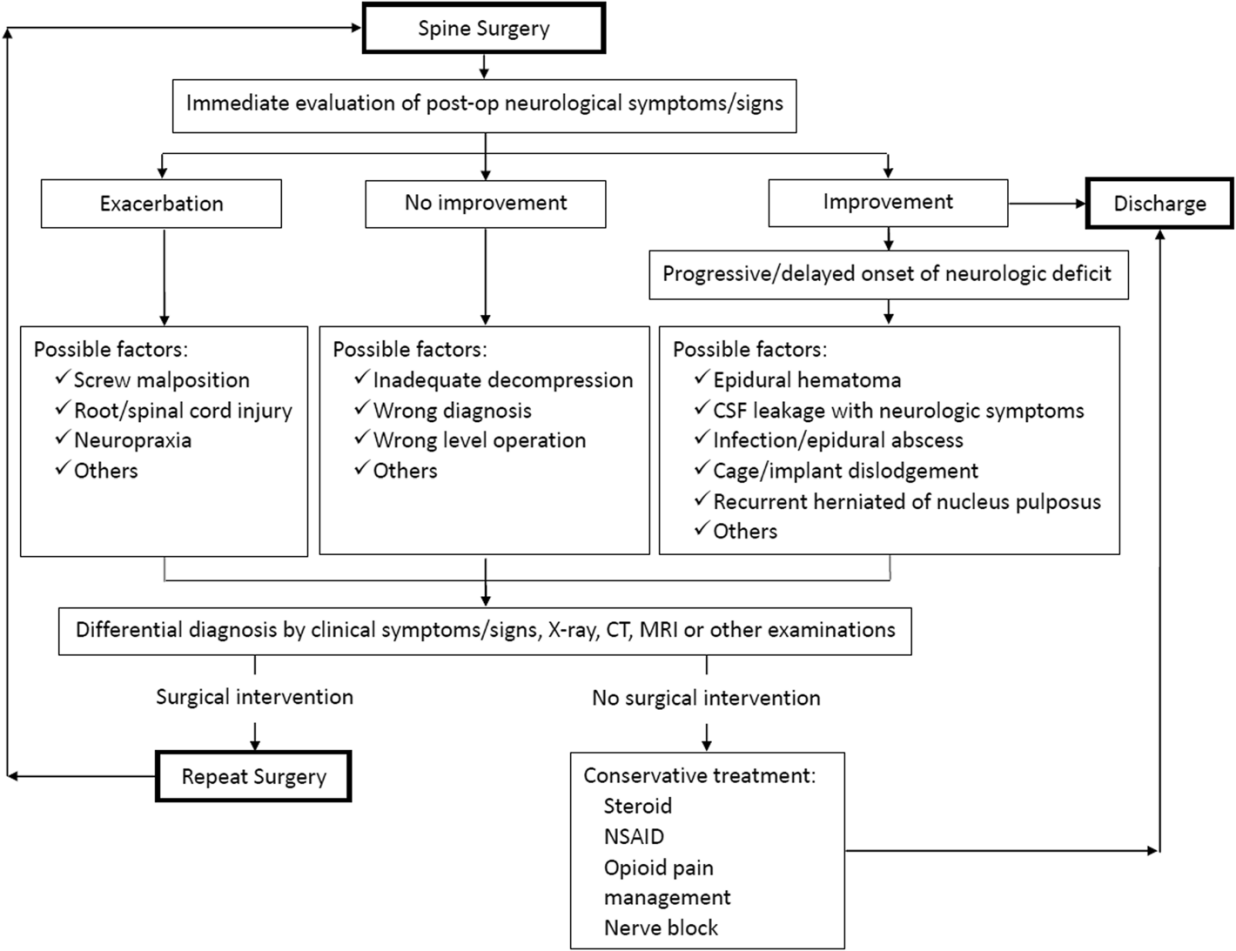
Post-spinal surgery flow chart to help determine possible causes of any required revision surgery. This flow chart is followed after a spinal surgery in our clinical practice to determine the possible causes of any revision surgery that may be required according to the evaluation of postoperative neurological symptoms and signs

In our clinical practice, we have proposed a post-spinal surgery flow chart to help surgeons determine the possible causes of any revision surgery that may be required (Fig. 4). The most important step is the immediate evaluation of postoperative neurological symptoms and signs. Postoperative exacerbation of neurological symptoms may be caused by screw malposition, spinal cord injury, or neuropraxia, which can be confirmed by radiographic assessment. If motor weakness and sciatica occurs as a result of a screw malposition, it can be clearly seen in plain radiographic imaging, and the misplaced screw(s) should be surgically revised immediately. However, if any screw malposition is radiographically equivocal, then a CT scan is indicated for further confirmation. If the symptoms are caused by spinal cord injury or neuropraxia, then conservative treatment is recommended because the patient would not benefit from revision surgery.

Epidural hematoma plays an important role in patients whose symptoms improve after operation but then experience the progressive onset of neurological deficits. Although there are other possible causes of postoperative neurological symptoms such as cerebral spinal fluid leakage, recurrent herniated nucleus pulposus and cage/implant dislodgement, a surgeon should inform patients the need of an emergent reoperation if they develop urgent symptoms such as motor weakness, decreased anal tone or urine retention, because the possibility of such symptoms resulting from symptomatic epidural hematoma is high and early revision surgery for removing epidural hematoma can result in better neurological outcomes. If the symptoms are less urgent, such as persistent lower limb numbness or saddle anesthesia, MRI with contrast can be used to help decide if revision surgery is needed.

If, after spinal surgery, there are persistent neurological symptoms that are nearly identical to the preoperative symptoms, then one should consider the possibility of inadequate decompression. Although MRI is informative in this regard, it is nonetheless very challenging for a surgeon to determine the presence or absence of inadequate decompression.

There were several limitations in this study, including possible selection bias because the patients were not randomized for retrospective observation. In addition, the cases included in the study all came from a single institution, which can lessen the generalizability of a retrospective analysis of this type. For example, a meta-analysis of 105 spine surgery complication studies conducted by Nasser et al. found that higher rates of complications were reported in prospective case studies versus retrospective cohort studies for cervical and thoracolumbar surgeries [26]. In addition, in some cases, medical chart data elements were missing, so complete data concerning patients’ neurological status was not obtained from all patients. Therefore, the neurological status of each patient was measured via improvements in muscle power, numbness sensation, and pain scale, instead of in the form of a thorough functional score. Furthermore, the choice of surgical procedure can vary greatly among different surgeons; for example, different surgeons may choose different implant systems and fusion levels for posterior instrumentation.

## Conclusions

An unplanned revision spinal surgery within 7 days after the initial operation can result in reduced quality of life, additional medical expenses, and patient distress, among other issues. In our institution, the overall incidence of unplanned revision spinal surgery between July 2004 and April 2011 was 1.12 %, with screw malposition, symptomatic epidural hematoma, and inadequate decompression being the most common surgical causes of reoperation within one week. Therefore, ensuring adequate decompression, preventing epidural hematoma, and proper pedicle screw placement may thus reduce the frequency of revision surgery.
